# Health-Seeking Behaviour for Childhood Ailments in Caregivers of Under-Five Children in an Urban Resettlement Colony in Delhi, India

**DOI:** 10.7759/cureus.24404

**Published:** 2022-04-23

**Authors:** Nandini Sharma, Saurav Basu, Subhanwita Manna, Pragya Sharma, Shivani Rao, Kushagr Duggal, Harpreet Kaur, Pawan Kumar, Shikha T Malik

**Affiliations:** 1 Community Medicine, Maulana Azad Medical College, New Delhi, IND; 2 Indian Institute of Public Health - Delhi, Public Health Foundation of India, New Delhi, IND; 3 State Health Intelligence Bureau, Directorate General of Health Services, New Delhi, IND; 4 Department of Biotechnology, National Biopharma Mission, Biotechnology Industry Research Assistance Council (BIRAC), New Delhi, IND

**Keywords:** antibiotic resistance, digital health, child care, child health, health seeking behaviour

## Abstract

This explorative qualitative study assesses the health-seeking behaviour for childhood ailments in caregivers of under-five children in a low-income neighbourhood in Delhi, India during July-September 2021. A total of 17 caregivers (mothers) of eight male and nine female under-five children were enrolled, with the mother being the caregiver in most (94%) cases.

Caregivers consulted on common childhood ailments from multiple sources, including family, neighbours, healthcare providers (both licensed and unlicensed), frontline workers, and local pharmacists. The internet was often used as a source of child health information due to its ease of access but often "confused" caregivers due to the presence of too much information.

Health-seeking behaviour of caregivers for childhood ailments could range from self-medication, local pharmacist dispensing, and private and public healthcare providers. Factors that influenced preference for the healthcare facility or provider were accessibility issues (waiting time, queuing), perceived physician competence, and associated out-of-pocket expenses. Caregivers reported dissatisfaction with government health facilities because of shorter operational hours, overcrowding, suboptimal sanitation, queuing with limited seating arrangements, and occasionally discourteous health staff. Self-medication and over-the-counter use of antibiotics was high due to a lack of awareness of the challenges of antibiotic resistance or any perceived side effects.

Preference for unlicensed practitioners for medical treatment was low and based on long-term familial beliefs and acceptance. However, traditional practitioners enjoyed a high level of trust in the community from shared cultural values, enjoining attenuation of the perceived non-biological agents of childhood illnesses through non-medical supernatural interventions.

## Introduction

India is home to nearly 158 million under-five children, comprising about 13% of its total population [1]. Under-five children in low-income countries continue to experience high morbidity and mortality due to factors such as low vaccination coverage, poor sanitation, increasing antimicrobial resistance, and undernutrition [2].

Apart from preterm complications and intrapartum events, the leading causes of under-five mortality are respiratory infections, diarrhoea, and other infectious causes, which are mostly preventable through childhood vaccination, sanitation, and optimal nutrition [3]. The United Nations promotes the twin goals of ending avoidable child deaths and promoting the health and well-being of children. The integrated management of newborn and childhood illnesses (IMNCI) strategies include the enhancement of healthcare accessibility, improved family and community practices, and effective case management of pneumonia, diarrhoea, malaria, measles, and malnutrition, conditions that account for an estimated 70% of the global burden of disease in the under-five population [4]. The National Family Health Survey-4 (2015-16), a large-scale representative nationwide cross-sectional survey in India, also observed a high burden of childhood morbidity from pneumonia, diarrhoea, and undernutrition [5].

Children, while susceptible to infections and undernutrition, are entirely dependent on their caregivers, usually their mothers, to obtain the necessary medical attention they require for the restoration of their health. Appropriate health-seeking behaviour by caregivers for their children can reduce morbidity and mortality resulting from most acute illnesses in childhood. However, despite the increasing focus on public health interventions to promote child health, neglect by caregivers due to poor awareness, false beliefs and perceptions regarding the disease, and lack of empowerment contribute to increased health risks for children. Such vulnerability is accentuated in communities with low maternal educational status, high burden of illiteracy, reduced healthcare accessibility, and household poverty, factors that aggravate disparities in their children's health-related awareness and practises [6]. Delayed healthcare-seeking for children's ailments despite the availability of affordable and accessible healthcare services contributes to preventable morbidity and mortality and enormous economic costs [7]. Furthermore, the importance of appropriate care-seeking is enhanced in resource-limited settings since healthcare providers can distinguish the severe cases needing referral and admission to higher facilities from those amenable to home-based management [8].

Caregivers may also resort to inappropriate administration of antibiotics to their sick children, either through self-medication or through unlicensed practitioners and pharmacists in settings where over-the-counter dispensing of antibiotics is highly prevalent, contributing to adverse effects, dysbiosis, and increased antimicrobial resistance in communities [9]. Caregiver interventions are also needed for the regular utilisation of free nutritional schemes and vaccination services for under-five children sponsored by the government of India for targeted reduction of undernutrition and susceptibility to infection [10,11].

The negative impact of the coronavirus disease 2019 (COVID-19) pandemic on the primary care system in India, especially during the lockdown, is likely to have further accentuated the challenge of healthcare accessibility for children due to limited outpatient department functioning, diversion of staff for COVID-19 testing and management, and parental fear of their children contracting COVID-19 [12]. However, there is a paucity of qualitative insights into the health-seeking behaviour of caregivers for their children in the context of the pandemic. 

Therefore, this study was conducted with the objective of assessing the health-seeking behaviour of caregivers of under-five children in a low-income neighbourhood in Delhi, India after the second wave of the COVID-19 pandemic. These observations would enable the identification of strategies for strengthening the primary health system services for under-five children and their utilisation by parents and caregivers in these resource-limited settings. 

## Materials and methods

Study design and setting

An exploratory qualitative study was conducted to critically explore the spectrum of health-seeking behaviour for childhood morbidities and concerns amongst caregivers of under-five children during the COVID-19 pandemic in Delhi, India from July 2021 to September 2021. The study location is a low-income urban agglomerate in the North-East district of Delhi (India), consisting of an urban resettlement colony and a slum area with an estimated 50,000 population. The primary care outpatient facilities available to the residents of the area included a Delhi government dispensary (DGD), a Delhi Jal Board dispensary, and an Aam Admi Mohalla Clinic apart from six to eight private licensed and unlicensed medical practitioners. The site was purposefully chosen as it is a demographic and health surveillance site for a government medical college in Delhi and affiliated postgraduate resident doctors provide routine maternal and child health services to the local population at the DGD. 

Sampling and study population

Primary female caregivers of under-five children who resided in the study setting for more than six months and had given birth in the previous five years were eligible to participate in the study. The study participants were selected through the simple random sampling method after obtaining the sampling frame from the baseline demographic survey. There was no non-response recorded in this study. A total of 17 caregivers were included until saturation of themes was observed, with no new codes occurring in the data. All the participants belonged to lower-middle socioeconomic status measured using the Kuppuswamy scale updated for the 2021 consumer price index [13]. 

Procedure

A semi-structured pretested interview guide was used to conduct qualitative in-depth face-to-face interviews with caregivers of under-five children to assess their health-seeking behaviour during acute childhood illnesses while considering a minimum recall period of six months. The interviews were conducted in the local language, Hindi, by two trained investigators, one male and one female. Throughout the interviews, the researcher was flexible and responsive, ensuring that participants were aware that they might end the session, leave, or refuse to answer any questions for any reason.

Data analysis

The interviews were audio-recorded, transcribed, and subsequently translated into English. The theory of planned behaviour (TPB) framework was adapted to construct a conceptual framework for understanding caregiver actions in response to childhood ailments and well-being [14]. Based on the TPB, attitudes (towards illness), subjective (cultural) norms, and perceived behavioural control (confidence in the ability to take corrective actions when encountering childhood ailments) were possible motivating factors determining the caregiver's health-seeking behaviour in response to various childhood illnesses. The following themes were identified in advance: "Health-seeking behavior for under-five children" and "Barriers to health-seeking behavior". 

The dataset was analysed using thematic analysis [15]. QDA miner lite (V 4.0) software was used to code the data. A sub-sample of transcripts was used to construct an initial list of descriptive codes and relevant quotes for each code. Three authors then went over the codes and discussed them until the final codes were agreed upon, and all subsequent transcripts were coded accordingly. New codes were added to the final set of codes when they emerged from the later transcripts. Using a pattern and focused coding process, final codes were reviewed again, categorised into groups, and finally into the thematic categories [15,16]. Relevant verbatim quotes were included to enhance the data interpretation while presenting the data. To maintain uniformity and quality, the consolidated criteria for reporting qualitative research (COREQ) guidelines [17] were followed throughout the study process.

Ethics

The study was approved by the Institutional Ethics Committee, Maulana Azad Medical College & Associated Hospitals, New Delhi (F.1/IEC/MAMC/84/02/2021/No385). Written and informed consent was obtained from all the participants. 

## Results

Participant characteristics

A total of 17 caregivers (mothers) of eight male and nine female under-five children were enrolled. The mean (SD) age of the female children was 20.5 (13.8) months, whereas that of the male children was 23 (12.5) months. The caregiver was the mother in most (94.1%) cases (Table 1).

**Table 1:** Socio-demographic characteristics of participants

Characteristic	Female (n=8) N, %	Male (n=9) N, %	Total (n=17) N, %
				Age of the child (months)			
0–18	4 (50.0%)	6 (66.7%)	10 (58.8%)
19–50	4 (50.0%)	3 (33.3%)	7 (41.2%)
Caregiver			
Mother	8 (100%)	8 (88.9%)	16 (94.1%)
Others	0	1 (11.1%)	1 (5.9%)
Educational status of caregiver			
No education	1 (12.5%)	2 (22.2%)	3 (17.6%)
Up to primary	1 (12.5%)	0 (0)	1 (5.9%)
Up to secondary	3 (37.5%)	2 (22.2%)	5 (29.4%)
High school/above	3 (37.5%)	5 (55.5%)	8 (47%)
Total family members			
0–4	4 (50.0%)	4 (44.4%)	8 (47.1%)
5–10	4 (50.0%)	5 (55.6%)	9 (52.9%)

A majority (58.8%) of the caregivers reported practicing exclusive breastfeeding until six months, and more than half (52.9%) also started weaning after six months of breastfeeding (Table 2).

**Table 2:** Health status and health-seeking behaviour of the participants BF, breastfeeding; ARI, acute respiratory infection; ICDS, Integrated Child Development Services Scheme.

Characteristics	Female child (n=8) N, %	Male child (n=9) N, %	Total (n=17) N, %
				BF status			
No BF	1 (12.5%)	4 (44.4%)	5 (29.4%)
Exclusive BF 6 months	7 (87.5%)	3 (33.3%)	10 (58.8%)
Predominantly BF	0 (0)	2 (22.2%)	2 (11.8%)
Bottle feeding			
Yes	5 (62.5%)	6 (66.7%)	11 (64.7%)
No	3 (37.5%)	3 (33.3%)	6 (35.3%)
Weaning status			
Not yet started	2 (25.0%)	0 (0)	2 (11.8%)
Before 6 months	0 (0)	3 (33.3%)	3 (17.6%)
At 6 months/later	6 (75.0%)	6 (66.7%)	12 (70.4%)
Diarrhoea			
Yes	6 (75.0%)	2 (22.2%)	8 (47.1%)
No	2 (25.0%)	7 (77.8%)	9 (52.9%)
ARI			
Yes	0 (0)	2 (22.2%)	2 (11.8%)
No	8 (100%)	7 (77.8%)	15 (88.2%)
Eye infection			
Yes	1 (12.5%)	0 (0)	1 (5.9%)
No	7 (87.5%)	9 (100%)	16 (94.1%)
Antibiotic course taken			
0	5 (62.5%)	5 (55.5%)	10 (58.8%)
1	2 (25%)	4 (44.5%)	5 (29.4%)
2–4	1 (12.5%)	0 (0)	1 (5.9%)
Knowledge of ICDS			
Yes	7 (87.5%)	8 (88.9%)	15 (88.2%)
No	1 (12.5%)	1 (11.1%)	2 (11.8%)
ICDS utilisation			
0–1	4 (50.0%)	2 (22.2%)	6 (35.3%)
2–4	4 (50.0%)	7 (77.8%)	11 (64.7%)

In our analysis, we identified the following major themes, discussed below with anonymised quotes in italics. Quotes and conversational trends are presented to support our identified themes.

Theme 1: "How We Do It": feeding practises for under-five children

Knowledge About Breastfeeding

Most mothers initiated breastfeeding within the first hour of giving birth and reported adherence to exclusive breastfeeding practises and breastfeeding on demand. Community health workers and local healthcare providers were considered trusted sources of information on breastfeeding. For instance, a participant reported that *"I was taking a few high dose medicines because of which doctor asked me not to breast feed, during that time I gave him bottle milk and now he doesn't drink my breast milk"* (P12, mother of a 1.5-year-old boy). 

Knowledge About Complementary Feeding

Most respondents stated that their children were started on complementary feeding at six months of age. The most frequent foods fed to children younger than nine months were dalia (porridge), *"daal ka pani"* (lentil soup water), roti, *"khichdi"* (rice-lentil preparations), and even glucose biscuits. Children aged over one year were often provided with food cooked for the family. Some of them preferred packaged items, such as *"Pediasure"* (formula baby food), because they were convenient and quick to prepare, but the drawbacks of processed foods were not yet understood by mothers. However, none of the mothers were aware of the concept of minimum dietary diversity attained through the consumption of at least five good groups per day by children between six and 23 months of age. 

Theme 2: "Where can we go": health-seeking behaviour of caregivers of under-five children

Source of Information

Caregivers consulted on common childhood ailments from multiple sources, including neighbours, relatives, healthcare providers, frontline workers, and pharmacists. Mothers believed that "professional" sources were more trustworthy and reliable. The internet was often used for sourcing information for childcare. 

*"We have technology in today's time, some information I get from the internet, some from my mother-in-law, some from the doctor and some from self, that's how should I take care of the baby, I observe things after all this".* (P3, mother of a 17-month-old boy). Another mother mentioned:* "I have downloaded an app MOMS and there all moms give their honest reviews, so we get an idea, also we have the elderly at home we ask them and reassure ourselves". *(P9, mother of a six-month-old girl).

In contrast, some mothers perceived the internet-sourced health information to lack credibility and authenticity. 

*"On internet I don't believe it in one go, I review comments and if they are positive only then I go with it. Also, I consult my sister-in-law and mother-in-law before doing anything for kids. There is a lot of information on the internet, which sometimes creates confusion"*. (P14, mother of a 1.5-year-old girl).

Health Seeking

Under-five children may experience ailments such as fever, cough, and diarrhoea. At the onset of such symptoms, most caregivers reported a tendency towards self-management with external health-seeking on persistence or deterioration of the condition. Health seeking could range from self-medication, local pharmacist dispensing, and private and public healthcare providers. *"I know the doctor will give this medicine to cure the fever of my child, so I gave the same medicine by directly getting it from the chemist shop. That's why you don't go to the doctor. If the fever or illness continues then I visit after 3 days, or else he gets fit and healthy within three days of taking the medicine".* (P12, mother of a two-year-old boy).

*"I have never taken him to a doctor either. He is the only child, so I take good care of him. He has only suffered from cold whenever there is a season change. Initially, when he started taking solid food, he used to have indigestion, gradually I understood with which food he was developing flatulence. So, I started giving food accordingly and he was fine. I have never made a doctor touch him at all. They have only given him immunization but touchwood no medicines were required at all. I feel there is an extreme rush in the dispensary (Primary Health Facility) and I don't feel comfortable there. I went there only when I conceived. And then went there for immunization".* (P8, mother of a 4.5-year-old boy).

Factors that influenced preference for the healthcare facility or provider were accessibility issues (waiting time, queuing), perceived physician competence, and out-of-pocket expenses. Private practitioners, according to most respondents, were convenient to access, especially during COVID-19. *"I usually went to nearby private clinics because it was corona going on and also the work is done quickly in a private hospital and there are a lot of crowds in a government hospital, and it takes a lot of time to get the medicines"*. (P14, mother of a two-year-old girl). In contrast, government health facilities had shorter operational hours and were overcrowded, limiting their utilisation. Lack of clean toilets, restricted seating areas, and occasionally discourteous health staff also contributed to dissatisfaction among respondents. 

Nevertheless, some mothers preferred government health facilities due to the perceived effectiveness of the treatment they received.

*"I feel the government one is better because their medicines work much better, the private hospital medicines take a lot of time to cure a disease".* (P16, mother of a 10-month-old boy).

*"I feel dispensary medicines are much better; they are light and effective; the private ones give heavier doses."* (P13, mother of a two-year-old girl). 

Preference for unlicensed practitioners was low and based on long-term familial beliefs and acceptance. 

*"My husband’s brother-in-law is a unlicensed doctor, so sometimes he used to ask him and bring medicine for him and he used to be ok. So, we never went to a licensed doctor for these things. He is a schoolteacher but he knows everything. He has a good knowledge about medicines".* (P16, mother of a 10-month-old boy).

Frontline workers were regarded as a reliable source of information by most caregivers due to their involvement with the community on childhood vaccination and common childhood illnesses.

Theme 3: Health-seeking barriers

Home-stocking of Medications

Most participants acknowledged keeping some drugs in their homes, including antibiotics, on a regular basis for common childhood ailments. Most participants could not distinguish between over-the-counter and prescription drugs due to the ubiquitous practice of local private pharmacies to dispense them indiscriminately even without a prescription. 

*"I have made a rule for my family that I keep medicines well in advance of basic illnesses like cough, cold, fever, loose motions, stomach-ache and I prefer not to go to the doctor and take medicine from home".* (P8, mother of 4.5-year-old boy).

Neutral Perceptions Towards Antibiotics

Awareness of antibiotics was low, although most could recognise some of the antibiotic suspensions and tablets that were commonly dispensed to children. Antibiotics were typically regarded as a *"specific drug,"* a *"harmful medicine,"* or a *"limited-use drug"*. One participant believed that antibiotics *"should not be given from the start".* None of the respondents were aware of the problem of antibiotic resistance. 

Supernatural Beliefs and Practices

A variety of traditional practitioners were consulted in the community by caregivers of young children, including *"Jhar phook wala"* (exorcists), tent doctors, healers, and godmen. A high degree of trust was achieved between traditional practitioners and some residents of the community due to shared cultural values, enabling resolution of the perceived non-biological realms of illness such as nazr, *"evil eye"*, that required non-medical supernatural interventions such as the application of protective enchantments. 

*"Medicines work on their part and taking down the evil eye work on its part. I feel both are required"*. (P11, mother of a 1.5-yr-old girl).

Furthermore, none of the caregivers voiced any major apprehension or fear of COVID-19 infection adversely impacting the child's health. 

## Discussion

This study examined the health-seeking behaviour and barriers to accessing under-five child health services in a Delhi urban resettlement colony. Like in previous studies, mothers comprised the principal decision-maker for child feeding with an inclination towards exclusive breastfeeding for the first six months after birth [18]. We observed a significant lack of awareness of the concept of dietary diversity with a preference for providing plant-based food as complementary feeding, which could be mostly due to the vegetarian ethos. According to Garg and Chadha (2009), egg and meat consumption was significantly lower in six- to eight-month-old babies in rural areas of Uttar Pradesh, India, due to parents' mistaken belief that their children would be unable to digest these meals [19]. Improving community awareness and acceptability of solids and even food of animal origin as complementary feeds for infants in families otherwise consuming non-vegetarian food, therefore, warrants consideration. 

Despite the growing tendency of sourcing health-related information from the internet, respondents were often confused about what constituted trustworthy and reliable digital sources due to the plethora of available information, suggesting the need for the formulation of community guidelines and awareness campaigns. Previous studies have indicated that health education programmes can help caregivers recognise high-burden childhood illnesses such as diarrhoea and pneumonia and promote healthcare-seeking while reducing their inappropriate self-medication practises [20-22]. 

In this study, mothers reported having decision-making power for deciding the health-seeking behaviour of their young children, in contrast to previous experiences in the developing world [23]. Barriers to effective health-seeking from public health facilities were an outcome of perceived negative attitudes and difficulty in accessing care in crowded conditions due to poor health system responsiveness, a factor observed in other resource-limited settings of the developing world [24,25]. A well-functioning health system, in theory, provides high-quality health services that satisfy patients and caregivers, resulting in increased demand for child healthcare services.

In this study, delayed health-seeking for childhood ailments was accentuated by adherence to supernatural beliefs and practices. Previous studies in developing world settings have suggested the power of cultural beliefs in influencing parental health-seeking behaviour of their children and the preference for a combination of biomedical and alternative treatments at the onset of illness [26,27]. Furthermore, in this study, the tendency to initially self-medicate the child in response to routine childhood illnesses was commonplace that likely interfered with early and appropriate care-seeking. Previous studies in low- and middle-income countries have also observed an increased likelihood of caregivers resorting to self-medication for treating perceived minor paediatric ailments that were consistent with their past experiences [28,29]. 

Finally, the very low burden of the severe acute respiratory syndrome coronavirus 2 (SARS-CoV-2) infection-associated complications and hospitalisation in the pediatric age group throughout the pandemic is likely to have significantly reduced caregiver concerns related to the potential effect of the infection upon their children [30]. 

The strength of the study is that it was conducted in an urban resettlement colony, which is representative of a large segment of the urban poor population in India and provides a fundamental description of a complex social reality. Study limitations include the inability to generalise the findings due to the qualitative design and the possible influence of the social desirability bias influencing participant responses. 

## Conclusions

The health-seeking behaviour of caregiver mothers of under-five children in a low-income neighbourhood was non-uniform and sourced from a variety of licensed and unlicensed practitioners, pharmacists, traditional healers, and frontline workers, apart from self-medication. Utilisation of government health facilities by caregivers for their children, except for immunisation services, was low due to low satisfaction with staff behaviour. Obtaining health-related information from internet-based sources is becoming more common, indicating the need for caregivers and young mothers to be educated on identifying trustworthy sources of health information.
